# A rare mutation causing autosomal dominant STAT1 deficiency in a South African multiplex kindred with disseminated BCG infection

**DOI:** 10.1186/s12887-023-04206-8

**Published:** 2023-07-29

**Authors:** Leonore Greybe, Daniel Leung, Nicole Wieselthaler, David M le Roux, Koon Wing Chan, Yu Lung Lau, Brian Eley

**Affiliations:** 1 Paediatric Infectious Diseases Unit, Red Cross War Memorial Children’s Hospital, Cape Town, South Africa; 2 Department of Paediatrics and Child Health, University of Cape Town, Cape Town, South Africa; 3 Department of Paediatrics and Adolescent Medicine, School of Clinical Medicine, Faculty of Medicine, Li Ka Shing, The University of Hong Kong, Hong Kong SAR, China; 4 Department of Radiology, University of Cape Town, Cape Town, South Africa

**Keywords:** BCG complications, BCG osteomyelitis, Disseminated BCG, STAT1 deficiency, STAT1 mutation, MSMD, Pediatrics

## Abstract

**Background:**

Autosomal dominant signal transducer and activator of transcription 1 (STAT1) deficiency, part of the Mendelian susceptibility to mycobacterial disease (MSMD) group, frequently causes disseminated Bacillus Calmette-Guérin (BCG) infections, but has not been reported from Sub-Saharan Africa (SSA) where routine birth BCG vaccination is practiced.

**Case presentation:**

Two half-siblings presented five years apart, with multifocal osteomyelitis as the dominant feature of disseminated BCG, which was successfully treated with antimycobacterial therapy. Whole exome sequencing demonstrated a novel heterozygous substitution in the splice site between intron 13 and exon 14 of the STAT1 gene, NM_007315: c.1128-1G>A, in the proband and his mother and was later confirmed in his half-brother.

**Conclusions:**

Children with BCG vaccine complications in SSA should be referred for further investigation and particular consideration of MSMD.

**Supplementary Information:**

The online version contains supplementary material available at 10.1186/s12887-023-04206-8.

## Background

Mendelian susceptibility to mycobacterial disease (MSMD) is a group of inborn errors of immunity (IEI) that impair the production and/or response to interferon-γ (IFN-γ), primarily resulting in susceptibility to weakly virulent mycobacteria including *Mycobacterium bovis* Bacillus Calmette-Guérin (BCG) and environmental mycobacteria. To date, mutations in 19 genes (*IFNG, IFNGR1, IFNGR2, STAT1, IL12B, IL12RB1, IL12RB2, IL23R, RORC, TBX21, IRF8, SPPL2A, ISG15, TYK2, JAK1, ZNFX1, NEMO, CYBB, USP18)* have been discovered that cause 35 different gene disorders [1, 2]. One of these disorders is autosomal dominant (AD) signal transducer and activator of transcription 1 (STAT1) deficiency (OMIM 614892), which frequently causes disseminated BCG infection [3].

Fifty four percent of patients with STAT1 loss-of-function (LOF) mutations present with MSMD at a median age of 1.3 years. In these patients, *M. bovis* BCG is the most frequently isolated microorganism and osteomyelitis a common clinical manifestation [4]. Antimycobacterial therapy is effective for the treatment of M. bovis BCG infection in STAT1 deficient patients. Primary and secondary prevention entails avoiding BCG vaccination in patients with STAT1 deficiency or in their family members until STAT1 deficiency has been excluded. There is currently no consensus recommendation for using secondary antimycobacterial prophylaxis in STAT1 deficiency patients after M. bovis BCG infection [4]. AD STAT1 deficiency has been reported in more than 25 patients, caused by 12 mutations, and affecting 4 of the 7 domains of the STAT1 protein [3, 5]. In Sub-Saharan Africa (SSA) where *Mycobacterium tuberculosis* is endemic and routine BCG vaccination at birth is generally practiced, disseminated BCG infection is seldom described, mainly in HIV-infected children [6]. According to our knowledge, the authors report the first AD STAT1 deficiency caused by a novel heterozygous mutation in a multiplex kindred in SSA.

### Case 1

**Fig. 1:**
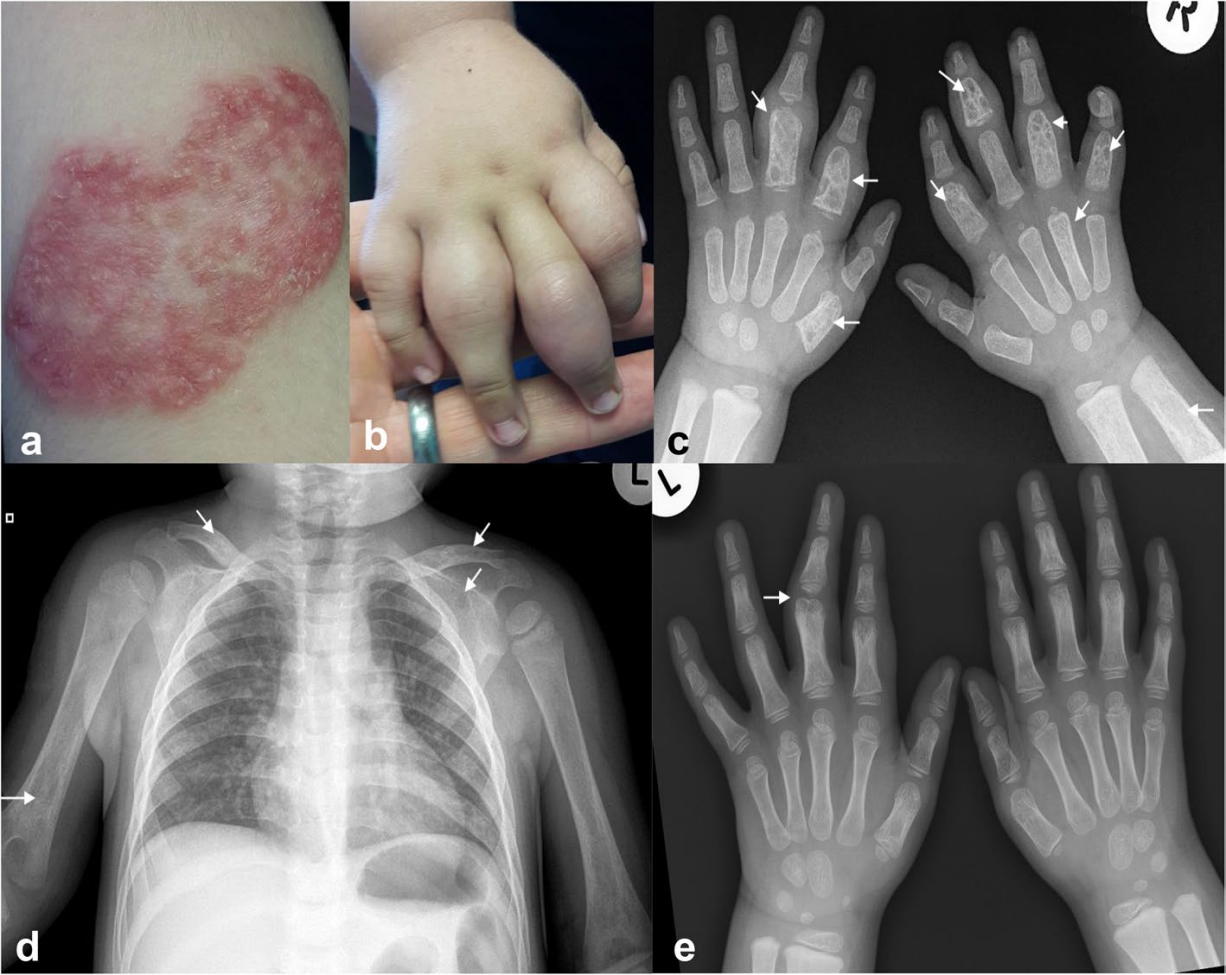
Clinical and radiological features of the proband. **a** Confluent non-healing plaque-like lesion, over the right deltoid muscle at the site of BCG vaccination, from which *Mycobacterium bovis* BCG was cultured. **b** Right hand, showing non-erythematous swelling over the 2nd, 3rd, 4th and 5th proximal interphalangeal joints. **c** AP radiograph of both hands demonstrates multifocal osteomyelitis with bone expansion and multiple ill- and well-defined lucencies of varying sizes affecting the left 1st metacarpal, left 2nd and 3rd proximal phalanges, right 2nd, 3rd, 4th and 5th proximal and middle phalanges with associated soft tissue swelling. The right 4th metacarpal and distal right radius and ulna shows bone expansion and small ill-defined lucencies. **d** Antero-posterior (AP) chest radiograph demonstrates multifocal osteomyelitis with bone expansion and ill-defined lucencies in the right distal humerus, both clavicles and left scapula. The heart and lungs are normal and there is no intrathoracic lymphadenopathy. **e** AP radiograph of both hands 2 years after presentation demonstrates interval resolution of the features of multifocal osteomyelitis. Healing deformity of left 3rd finger

The proband, a previously well, HIV uninfected, 2-year-old boy born to nonconsanguineous parents presented with chronic erythematous, papular skin lesions over the right deltoid muscle at the site of BCG vaccination and on the right side of his chest wall, and bilateral finger swelling. BCG vaccination at birth was followed by the development of a nodule at the vaccination site surrounded by a cluster of raised papules which over time coalesced to form an erythematous, non-healing plaque-like lesion without fistulation (Fig. 1a). At 18 months of age, he developed a similar cluster of papules on the right anterior chest wall, inferolateral to the right nipple. Additionally, he also developed finger swelling (Fig. 1b) around the same time. Imaging of the hands and chest revealed multiple lucencies of phalanges of both hands, the right distal humerus, both clavicles and left scapula, consistent with multifocal osteomyelitis (Fig. 1c, d). Skin biopsies of the BCG site and chest wall lesions cultured *Mycobacterium tuberculosis* complex, which on molecular testing was confirmed to be BCG, susceptible to both rifampicin and isoniazid. Immunological evaluation revealed raised IgG, IgM, and IgA concentrations, normal CD3, CD4, CD8, CD19 and CD16/56 cell counts, and a normal oxidative burst test (dihydrorhodamine 123 (DHR) as oxidative probe) – refer Supplementary file. He was started on four-drug antimycobacterial therapy (rifampicin, isoniazid, ethambutol, and levofloxacin), after 3 months ethambutol and levofloxacin were discontinued. Rifampicin and isoniazid were stopped after 18 months. He is currently 9 years old and has remained asymptomatic for 5 years. He has not experienced further serious illness such as salmonella or candida infection, mycobacterial infection, or hospitalization. He has a mild residual deformity of the middle finger of his left hand without any functional consequences (Fig. 1e).

### Case 2

**Fig. 2:**
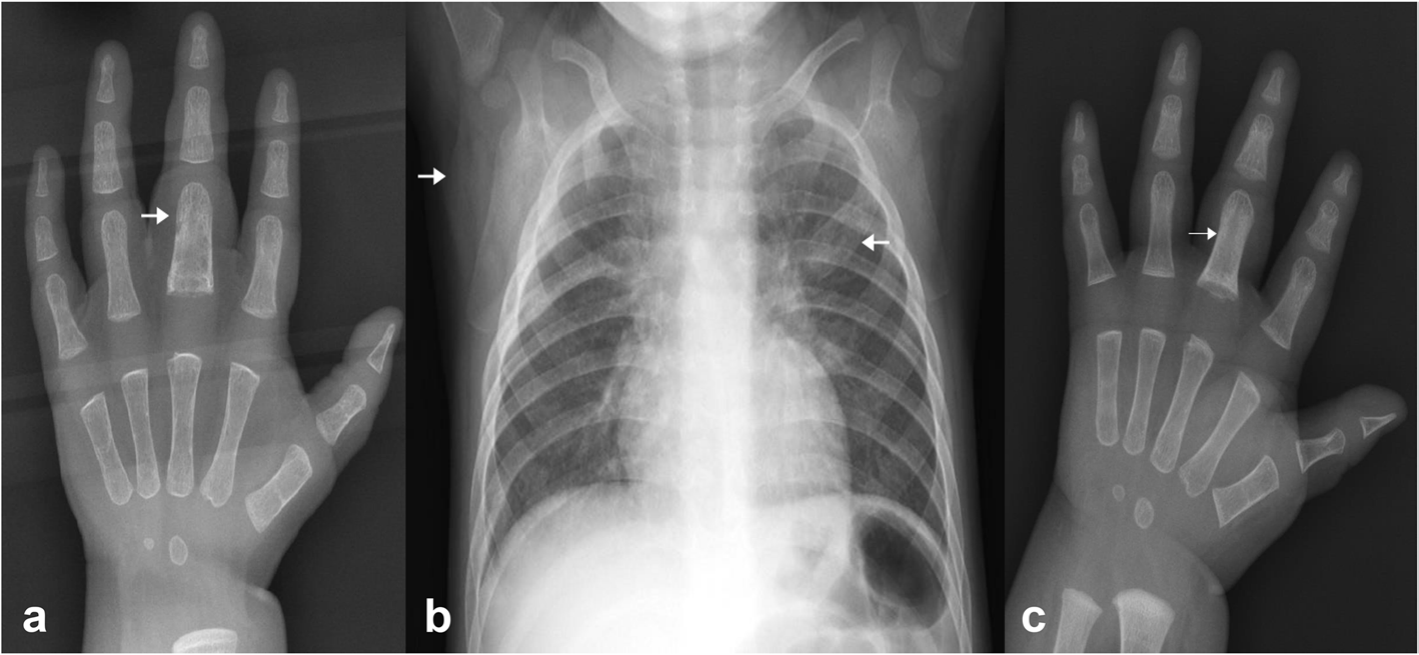
Radiological images of the proband’s half-brother. **a** AP radiograph of the right hand demonstrates expansion of the proximal 3rd phalanx with multiple ill-defined lucencies consistent with osteiomyelitis. There is associated soft tissue swelling. **b** AP chest radiograph demonstrates right axillary soft tissue swelling consistent with adenopathy. There is expansion of the 2nd left anterior rib with ill- defined lucencies consistent with osteomyelitis. Lateral x-ray confirmed the absence of mediastinal adenopathy. **c** AP radiograph done after 4 months demonstrates improvement of the soft tissue swelling, decreased expansion and sclerosis of the 3rd proximal phalanx consistent with healing osteomyelitis

Five years after the proband presented, his well-grown, HIV uninfected, 10-month-old, maternal half-brother presented with intermittent swelling and decreased range of motion of the right middle finger and the right knee. He received BCG vaccine at birth and developed ulceration at the BCG vaccination site with surrounding papule formation and swollen right axillary lymph nodes. At the time the local clinic prescribed a course of antibiotics. At presentation, he had a healing scar at the BCG vaccination site, a small single firm lymph node with soft tissue swelling in the right axilla, uniform soft tissue swelling of the right middle finger, and normal lower limbs. Radiology showed expansion of the proximal 3rd phalanx with multiple ill-defined lucencies (Fig. 2a), and expansion of the 2nd left anterior rib with ill- defined lucencies consistent with multifocal osteomyelitis. Right axillary soft tissue swelling consistent with adenopathy was demonstrated. Lung fields were clear, and there was no mediastinal adenopathy (Fig. 2b).

Blood culture yielded no organisms. There were no skin lesions or lymph nodes amenable to biopsy. Immunological evaluation included normal levels of IgG, IgM, and IgA concentrations, normal CD3, CD4, CD8, CD19 and CD16/56 cell counts, and a normal (DHR) oxidative burst test – refer Supplementary file. A presumptive diagnosis of disseminated BCG infection with multifocal osteomyelitis was made and rifampicin, isoniazid, ethambutol, and levofloxacin were commenced. After three months he was stepped down to rifampicin and isoniazid. Repeat x-ray demonstrated healing osteomyelitis (Fig. 2c). He is now 3 years old, has completed 18 months of antimycobacterial therapy, is well, and has not experienced recurrent mycobacterial infection or other serious infections.

### Mutational analysis

**Fig. 3:**
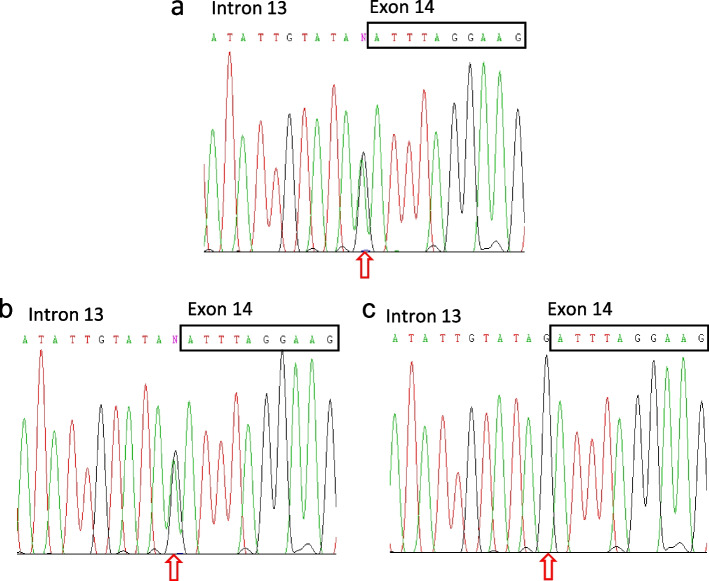
Sanger sequencing validation chromatogram of the proband parents showing the mutation, c.1128-1G>A (IVS13-1G>A), affecting the intron 13/exon 14 splice junction (intron 13 acceptor site). **a** Proband, with arrow indicating the position of the heterozygous c.1128-1G>A mutation. **b** Proband’s mother with arrow indicating the position of the heterozygous c.1128-1G>A mutation. **c** Proband’s father, with arrow indicating no mutation

**Fig. 4:**
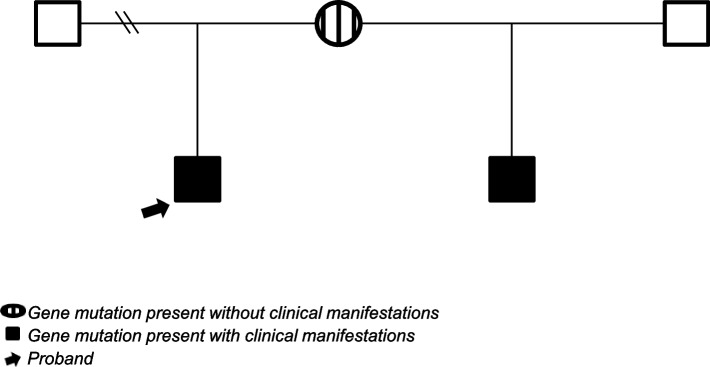
Genogram consistent with autosomal dominant inheritance

Whole exome sequencing demonstrated a novel heterozygous substitution in the splice site between intron 13 and exon 14 of the STAT1 gene, NM_007315: c.1128-1G>A, in the proband and his mother but not in his father. The mutation was confirmed in both the proband and his mother by Sanger sequencing (Fig. 3). The identical heterozygous mutation was recently confirmed in his half-sibling. The biological father of the half-sibling was not tested and there was no other maternal offspring (Fig. 4).

## Discussion and conclusion

Multifocal osteomyelitis was a striking feature of disseminated BCG infection in both half-siblings. They were otherwise remarkably well at the time of diagnosis. Multifocal osteomyelitis may be a feature of disseminated BCG infection in patients with AD IFN-γ receptor 1 (IFN-γR1), and AD STAT1 deficiencies, and if present distinguishes these IEI from other MSMD disorders [7].

The rare mutation identified in our patients, c.1128-1G>A, is unreported on public genome and mutation databases including gnomAD and CLIN Var; and is classified as pathogenic according to American Academy of Allergy Asthma and Immunology guideline 2020 [8]. Furthermore, a patient in China with AD STAT1 deficiency manifesting with multifocal osteomyelitis and carrying a mutation c.1128-2A>G in the same splice site that impairs the IFNγ response, was recently identified further supporting that a mutation in this splice site will be pathogenic [9].

Despite the presence of the mutation and receipt of the BCG vaccine at birth, the mother, born in 1981, has not yet developed mycobacterial infection. Incomplete penetrance is documented in AD STAT1 deficiency and may explain why this mother has not experienced mycobacterial infection [7]. An alternative explanation for not developing BCG infection is that the BCG vaccine that she received was incorrectly administered. Between 1973 and 2000 South Africa administered BCG vaccine to newborns by the percutaneous route using a multi-puncture tool that was repeatedly reused after autoclaving. A study published in 1995 showed that there was little evidence of skin penetration with this tool, suggesting that many children were inadequately inoculated during BCG vaccination [10].

The occurrence of BCG osteomyelitis in the maternal half-brother was unfortunate as BCG vaccination was received despite the mother carrying a letter advising against BCG vaccination in subsequent siblings. This highlights the importance of raising awareness and educating vaccine providers about IEI where live vaccines will be contraindicated.

In SSA where neonatal BCG vaccination is widely practised, disseminated BCG infection has been infrequently described, mainly in HIV-infected children [6, 11]. In HIV-uninfected children, severe combined immunodeficiency, chronic granulomatous disease and MSMD have been reported to present with disseminated BCG infection in SSA [11–13]. However, the small number of patients in reports from SSA is currently insufficient to establish the most frequent IEI associated with disseminated BCG infection in this region [11–13]. Reasons for the scarcity of SSA reports primarily relate to limited laboratory capacity for diagnosing both BCG infection and IEI.

Disseminated BCG infection should always alert the clinician to the possibility of HIV-infection or IEI. A tiered approach to investigations starts by excluding HIV infection, and assessing the full blood count and differential count results which are accessible in most primary healthcare settings. Second-line testing include immunoglobulin isotype and lymphocyte subset concentrations and neutrophil function assessment. The QuantiFERON-TB-Plus, can additionally be used as an unvalidated screening test of the functional IFN-γ pathway in the absence of additional testing. Molecular testing should be considered in consultation with a pediatric immunologist [12, 14].

In African countries where these capacities exist, this report should encourage clinicians and researchers to refer children with BCG vaccine complications for further investigation and consideration of IEI, particularly MSMD.

## Supplementary Information

**Additional file 1: Suppl. Table 1.** Total immunoglobulin and lymphocyte subset concentrations of the maternal half-brothers with BCG osteomyelitis.

## Data Availability

Data analysis was not performed for this case report.

## Abbreviations

MSMD: Mendelian susceptibility to mycobacterial disease
IEI: Inborn errors of immunity
IFN-γ: Interferon-γ
BCG: Bacillus Calmette-Guérin
AD: Autosomal dominant
STAT1: Signal transducer and activator of transcription 1
SSA: Sub-Saharan Africa
HIV: Human immunodeficiency virus
DHR: Dihydrorhodamine
